# Parental knowledge of alcohol consumption: a cross sectional survey of 11–17 year old schoolchildren and their parents

**DOI:** 10.1186/1471-2458-13-412

**Published:** 2013-04-30

**Authors:** Michela Morleo, Penny A Cook, Gill Elliott, Penelope A Phillips-Howard

**Affiliations:** 1 Centre for Public Health, Liverpool John Moores University, Henry Cotton Campus (second floor), 15-21 Webster Street, Liverpool L3 2ET, UK; 2 School of Health Sciences, University of Salford, Allerton Building, Salford M6 6PU, UK; 3 Faculty of Education, Edge Hill University, St Helens Road, Ormskirk, Lancashire L39 4PQ, UK; 4 Liverpool School of Tropical Medicine, Pembroke Place, Liverpool L3 5QA, UK

**Keywords:** Alcohol, Parents, Adolescents

## Abstract

**Background:**

Developing timely and effective strategies for preventing alcohol misuse in young people is required in order to prevent related harms since, worldwide, alcohol consumption was associated with 320,000 deaths amongst 15–29 year olds in 2004. Providing guidance and advice to parents is essential if alcohol misuse is to be reduced. However, prevention of risky behaviours is hampered if parents are unaware of the risks involved.

**Methods:**

A cross-sectional school-based survey of parent–child dyads, simultaneously questioning 935 children aged 11–17 years old and their parent(s). Univariate and multivariate associations are reported between demography, alcohol behaviours and parental knowledge of their child’s alcohol consumption.

**Results:**

41.1% (n = 384) of children reported drinking alcohol. Of these, 79.9% of their parents were aware of their child’s alcohol consumption. Children aged 11–14 years had over a twofold greater odds of consuming alcohol without parental knowledge compared with 15–17 year olds (AOR: 2.7, 95% CI: 1.3-5.7). Of parent–child dyads where the child reported consuming alcohol, 92.7% of parents reported that they had spoken to their child about alcohol at least once in the past three months, whereas 57.3% of their children reported that this had occurred. Children who consumed alcohol and whose parents did not know they drank alcohol were less likely to report having a parental discussion about alcohol in the last three months (AOR: 0.4, 95% CI: 0.1-1.0) or report lifetime receipt of at least one other parenting protective measure (AOR: 0.5, 95% CI: 0.2-0.9) compared with those children who drank alcohol with parental knowledge.

**Conclusions:**

Whilst only small numbers of young adolescents in our sample were drinking alcohol compared with older adolescents, those who did were more likely to do so without their parents’ knowledge. These two factors combined (drinking earlier and drinking without parental knowledge) could place children at risk of immediate harm. Further research is essential to identify whether public health strategies should be developed which could support parents to employ lifestyle parenting techniques even before the parent believes the child to be at risk.

## Background

Worldwide, alcohol consumption was associated with 2.5 million deaths in 2004 [1,2]. Half of these deaths were related to liver cirrhosis. However, 320,000 represented deaths in young people aged 15–29 years, for whom the acute consequences of alcohol misuse (such as alcohol-related road traffic accidents) can be more common [3]. Whilst alcohol consumption generally amongst young people in England is thought to be declining, [4-6] evidence suggests that consumption patterns remain high amongst a minority [4,6]. These individuals are at risk of a range of harms including violence and regretted/unprotected sex [7-11], hospital admission [12,13] and neurological damage [14]. Those whose alcohol careers begin at a young age are at a heightened risk of long term harm [15,16]. This is of particular concern given the observed trends towards younger alcohol initiation [3].

Developing timely and effective strategies for preventing alcohol misuse in young people is essential in order to prevent related harms. Over 100 countries worldwide have banned alcohol consumption and/or purchase by children (in off and/or on-licensed premises) [17]. Typically, the minimum age for doing so is 18 years old. Despite this, considerable proportions of underage young people access alcohol regularly and in large quantities [7,8], using a range of methods (including through parents, illegal self-purchase, friends, and adult strangers), one of the most common ways being through their parents [18,19]. Large scale surveys in the United States of America (USA) and one region in England have found that acquiring alcohol from parents appeared to offer some protection against children’s harmful drinking behaviours such as binge drinking, frequent drinking, alcohol-related violence and alcohol-related regretted sex [7,20-22]. However, this is not universal and other surveys in the USA suggest that provision of alcohol at parties and to those aged 12 years are associated with increased alcohol consumption/abuse [22,23]. Providing information to parents on methods used to obtain alcohol by underage children and the impacts of this is essential if alcohol misuse is to be reduced. In the UK, guidelines have been produced advising parents that children under 18 years should not drink, but if they do, to reduce the risks involved through reducing the quantities of alcohol that young people consume and frequency with which they consume it [24]. However, the prevention of risky behaviours (such as binge drinking and alcohol-related violence) is hampered if parents are unaware of the risks involved [25].

Internationally, a number of studies have investigated parental knowledge of alcohol consumption by their children. Research in New York City (USA) has suggested that up to a fifth of parents of female sixth graders (aged 11–12 years) may be unaware of their child’s alcohol consumption, and awareness of risks faced may impact on parenting techniques used [25]. However, parent–child dyad research has tended to focus on either one gender or one age group in relation to the child [25-30], and so has been unable to explore the different characteristics of young people whose parents are aware of their alcohol consumption compared with those who are not. Further, research published to date has not explored the role of socio-economic status (SES) on parental knowledge [25-30], although this has a significant relationship with likelihood of alcohol consumption and how children access alcohol [7]. Using parent–child dyads, this paper explores the characteristics (including SES) of children (aged 11-17 years, those under the typical international legal drinking age [17]) who consume alcohol with or without parental knowledge, how they access alcohol, experiences of potential harm (here measured by drunkenness), and parenting methods employed.

## Methods

The term ‘child’ is used throughout this paper to describe the schoolchildren, aged between 11 and 17 years, since this better describes the link between parents and their offspring than other descriptors such as ‘young person’. Four schools in Wirral (northwest England, which has one of the highest rates of alcohol-related hospital admission for those aged under 18 in England [12]) participated in the survey across 14 school events held over two academic years (September to August: 2008/09 and 2009/10). The schools represented a combination of state/independent (two independent and two state schools), mixed/single sex schools (one single sex and three mixed schools from a total of 25 secondary schools in the area) and catered for different age ranges (two were for 3–19 year olds, one for 11–16 year olds and one for 11–18 year olds). In total, during 2010, 3,704 pupils attended the four schools (3–19 years) [31]. Researchers used convenience sampling to recruit secondary age pupils (11–17 years) and their parents to the study at school review days and parent evenings where both the child and their parent were present at the school simultaneously. Both were provided with verbal and written information about the study; parents were asked to provide informed consent for themselves and for their child, and children aged over 16 years were asked to provide informed consent for themselves. Children and their parent(s) were requested to self-complete their questionnaire in private away from the other family member. Questionnaires were coded to enable dyad responses to be linked.

Researchers approached 1,313 parent/child dyads for recruitment into the study; 90.0% (n = 1,180) agreed to participate (31.9% of the total number of pupils attending the sampled schools). In total, 216 questionnaires were excluded from analysis due to: no parent/child dyad linkage, incomplete core data items (such as demographic indicators or consumption of alcohol), or age of the child exceeding the inclusion criterion (over 18 years). Questionnaires were also excluded if the carer present at the school was not the mother, father or step-parent of the child (n = 29). The total sample available for analysis was 935 dyads.

Questionnaire topics for the child included: demographic details (age, gender, postcode); consumption of alcohol (yes/no); main method of obtaining alcohol (parents, friends, shop, other); experience of past month drunkenness (yes/no/don’t know); experience of recent (past three months) discussions with parents about alcohol (yes/no); and reported parenting strategies associated with alcohol use (yes/no). The parents’ questionnaire followed a similar format, including parental relationship to child (mother, father, step-parent, other); perception of whether their child consumed alcohol (yes/no); their child’s experience of past month drunkenness (yes/no/don’t know); whether they had talked to their child about alcohol in the last three months (referred to as recent alcohol discussions, yes/no), and lifetime prevalence of other parenting techniques. For lifetime prevalence of other parenting techniques, both schoolchildren and parents were asked if the parent had ever used any of the following techniques to prevent and/or reduce alcohol use (yes/no): grounding the child or removing privileges; provision of small quantities of alcohol by the parent; stopping the child from seeing certain friends; controlling pocket money or income; hiding or reducing the amount of alcohol in the house; organising lifts to/from parties; parents reducing their own drinking; parents ensuring their child had a mobile phone; and other (undefined). The similarity in questionnaire design allowed comparison between parents’ perceptions of their child’s experiences and their child’s actual reported experiences. For analysis, responses to these questions were aggregated into the derived binary variable “lifetime use of at least one parenting technique”. Where parents reported not knowing whether their child had been drunk in the past month, this was classified as no knowledge of drunkenness. Where children reported not knowing that they had been drunk in the past month, this was classified as not having been drunk.

Postcodes of place of residence reported by each dyad (n = 727) were assigned to their resident Lower Super Output Area (LSOA; geographical areas with an average population of approximately 1,500 individuals), which was in turn assigned to their 2007 Index of Multiple Deprivation (IMD) score and quintile [19,32]. (IMD is a national measure based on income, employment, skills and training, and barriers to housing, assigned to LSOA.)

Data entry, screening, coding and analysis were completed in SPSS version 17. Ethical approval for the study was received from the Ethical Committee of Liverpool John Moores University. Univariate and multivariate associations are reported between demographic details (child’s age, gender, ethnicity, resident deprivation quintile and school), and the analysis variables (children who drink alcohol, children who drink alcohol without parental knowledge, child’s main method of obtaining alcohol, child’s past month drunkenness, parent reports of recent alcohol discussions, parental reporting of lifetime use of other parenting techniques). After the initial analyses investigating children who drink alcohol, the ethnicity variable was excluded from all subsequent statistical models because the majority of children belonged to one ethnic group. Parental relationship to their child showed no significant associations and so was also excluded from the model. Parental knowledge of their child’s alcohol consumption was added as a covariate for binary logistic regression analyses to predict child’s main method of obtaining alcohol, child’s past month drunkenness, parental reports of recent alcohol discussions, and parental reports of lifetime use of other parenting techniques. Multinomial logistic regression was used to find predictors of children’s main method of obtaining alcohol. There were four options for this outcome variable: parents, friends, shop, other. Acquiring alcohol from parents was used as the reference category because it was the most common method young people used to access alcohol in our sample and generated the most stable coefficient estimates. The classification is slightly ambiguous because we did not measure whether the acquisition of alcohol from parents was done with parental consent; however, we also had evidence from two studies that used the same question that a positive response to this was protective [18,19]. Predictor variables were children’s demographic details and parental knowledge of alcohol consumption.

## Results

Over two-thirds of parental respondents were mothers (71.4%), 20.2% fathers, 6.8% both parents and the remainder (1.5%) step-parents. Of the 935 children recruited, 67.7% were male, and 63.7% were aged 11 to 14 years old (Table 1). Of those for whom ethnicity was available (n = 882), 96.9% were white and of those for whom deprivation quintile was available (n = 727), 35.4% resided in the most deprived quintile. In total, 41.1% of children reported consumption of alcohol. In univariate analyses, alcohol consumption was significantly associated with age, ethnicity, deprivation and school, but not gender. For example, younger schoolchildren were less likely to consume alcohol than their older peers (24.8% of 596 11–14 year olds drinkers; 95% CI 21.4-28.5; 69.6% of 339 15–17 year old drinkers, 95% CI 64.4-74.5; Table 1). In multivariate analysis, controlling for demographic and other factors (age, ethnicity, gender, deprivation and school), pupils aged 15–17 years had a tenfold higher odds of consuming alcohol compared with 11–14 year olds (adjusted odds ratio, AOR, 10.4, 95% CI 6.8-15.8). Children who consumed alcohol had a fourfold higher odds of residing in the most affluent quintile compared with those in the most deprived quintile (AOR 4.3, 95% CI 2.0-9.0).

**Table 1:** Characteristics of children (aged 11–17 years old) associated with drinking alcohol

	**df**	**n**	** Univariate**		** Multivariate**	
			**% (95% CI)**	**P**	**AOR (95% CI)**	**P**
Child’s gender						
Male	1	633	40.4% (36.5-44.4)	0.572	Ref	0.121
Female		302	42.4% (36.7-48.2)		1.5 (0.9-2.6)	
Child’s age						
11-14	1	596	24.8% (21.4-28.5)	<0.001	Ref	<0.001
15-17		339	69.6% (64.4-74.5)		10.4 (6.8-15.8)	
Child’s ethnicity						
White	1	855	41.4% (38.1-44.8)	0.046	3.3 (1.0-10.2)	0.042
Other		27	22.2% (8.6-42.3)		Ref	
Deprivation quintile						
1 (most affluent)	4	61	62.3% (49.0-74.4%)	<0.001	4.3 (2.0-9.0)	0.005
2		126	40.5% (31.8-49.6)		1.2 (0.7-2.0)	
3		124	47.6% (38.5-56.7)		1.5 (0.9-2.5)	
4		159	38.4% (30.8-46.4)		1.2 (0.7-1.9)	
5 (most deprived)		257	31.9% (26.3-38.0)		Ref	
School						
School A	3	196	31.6% (25.2-38.6)	<0.001	Ref	0.286
School B		192	34.9% (28.2-42.1)		1.6 (1.0-2.8)	
School C		118	55.1% (45.7-64.3)		0.9 (0.4-2.0)	
School D		429	44.3% (39.5-49.1)		1.1 (0.6-1.8)	
Total		935	41.1% (37.9-44.3)			

Ref refers to the reference category.

Of children who reported alcohol consumption (n = 384), 79.9% of parents were aware that their child had consumed alcohol. If younger children were consuming alcohol, they were more likely to be doing so without their parents’ knowledge (35.1% of 148 11–14 year old drinkers, 95% CI 27.5-43.4; compared with 10.6% of 236 15-17 year old drinkers, 95% CI 7.0-15.2, Table 2). This was confirmed using logistic regression after controlling for confounding factors such as gender, age, socio-economic status and school (AOR 2.7, 95% CI 1.3-5.7). There was no statistically significant association found between lack of parental knowledge of alcohol consumption and their socio economic status or the child’s gender.

**Table 2:** Characteristics of drinking children (aged 11–17) associated with no parental knowledge of their consumption

	**df**	**n**	**Univariate**		**Multivariate**	
			**% (95% CI)**	**P**	**AOR (95% CI)**	**P**
Child’s gender						
Male	1	256	17.6% (13.1-22.8)	0.087	Ref	0.135
Female		128	25.0% (17.8-33.4)		0.5 (0.2-1.2)	
Child’s age						
11-14	1	148	35.1% (27.5-43.4)	<0.001	2.7 (1.3-5.7)	0.007
15-17		236	10.6% (7.0-15.2)		Ref	
Deprivation quintile						
1 (most affluent)	4	38	21.1% (9.6-37.3)	0.581	0.6 (0.2-1.9)	0.837
2		51	15.7% (7.0-28.6)		0.7 (0.3-1.8)	
3		59	22.0% (12.3-34.7)		0.6 (0.3-1.6)	
4		61	18.0% (9.4-30.0)		0.9 (0.3-2.1)	
5 (most deprived)		82	26.8% (17.6-37.8)		Ref	
School						
School A	3	62	17.7% (9.2-29.5)	<0.001	Ref	0.005
School B		67	52.2% (39.7-64.6)		3.2 (1.1-9.1)	
School C		65	20.0% (11.1-31.8)		2.0 (0.6-7.3)	
School D		190	9.5% (5.7-14.6)		0.4 (0.2-1.2)	
Total		384	20.1% (16.2-24.4)			

Ref refers to the reference category.

The most prevalent method of obtaining alcohol was from the parents, followed by the child’s own friends (48.4% and 23.7% of drinkers respectively). Univariate analysis indicated associations between source of alcohol and gender; for example, a higher percentage of males than females obtained most of their alcohol from their parents (53.7%, 95% CI 46.8-60.6, compared with 37.3% among females, 95% CI 27.9-47.4), while females were more likely than males to mainly obtain alcohol through their friends (37.3%, 95% CI 27.9-47.4, compared with 17.3% among males, 95% CI 12.5-23.0; Table 3). Using multinomial regression with main method of obtaining alcohol as the outcome (and using obtaining alcohol from parents as a reference category), girls (compared with boys) and older pupils (compared with younger pupils) were significantly more likely to obtain alcohol through their friends than from their parents, after accounting for demographic factors and parental knowledge of alcohol consumption (girls compared with boys: AOR 6.9, 95% CI 1.8-26.2; 15–17 year olds compared with 11–14 year olds: AOR 3.1, 95% CI 1.3-7.7). Younger pupils had a significantly lower odds of obtaining alcohol from a shop than from their parents compared with older pupils (11–14 year olds compared with 15–17 year olds: AOR 0.2, 95% CI 0.0-0.6). Those who consumed alcohol without parental knowledge of their drinking had over a threefold higher odds of mainly obtaining alcohol through their friends than from their parents, compared with children who consumed alcohol with parental knowledge (AOR 3.6, 95% CI 1.4-9.3).

**Table 3:** Main method of obtaining alcohol for drinkers (11-17 years) using chi square and multinomial logistic regression

		**df**	**n**	Parents*	**Friends**		**Shop**		**Other**	
				**% (95% CI)**	**% (95% CI)**	**AOR P**	**% (95% CI)**	**AOR P**	**% (95% CI)**	**AOR P**
					**AOR (95% CI)**		**AOR (95% CI)**		**AOR (95% CI)**	
Gender	Male	3	214	53.7% (46.8-60.6)	17.3% (12.5-23.0)		15.0% (10.5-20.4)		14.0% (9.7-19.4)	
					0.1 (0.0-0.6)	0.005	0.3 (0.1-1.3)	0.112	0.4 (0.1-1.6)	0.194
	Female		102	37.3% (27.9-47.4)	37.3% (27.9-47.4)		13.7% (7.7-22.0)		12.0% (6.4-20.0)	
					Ref		Ref		Ref	
Age	11-14	3	108	56.5% (46.2-66.0)	22.2% (14.8-31.2)		8.3% (3.9-15.2)		13.0% (7.3-20.8)	
					0.3 (0.1-0.8)	0.013	0.2 (0.0-0.6)	0.008	0.6 (0.2-1.6)	0.287
	15-17		208	44.2% (37.4-51.3)	24.5% (18.8-30.9)		17.8% (12.8-23.7)		13.5% (9.1-18.9)	
					Ref		Ref		Ref	
Deprivation quintile	1 (most affluent)	12	31	38.7% (21.8-57.8)	38.7% (21.8-57.8)		12.9% (3.6-29.8)		9.7% (2.0-25.8)	
					2.3 (0.7-8.3)	0.189	1.7 (0.4-7.6)	0.504	2.6 (0.5-14.4)	0.259
	2		42	52.4% (36.4-68.0)	26.2% (13.9-42.0)		7.1% (1.5-19.5)		14.3% (5.4-28.5)	
					1.7 (0.6-4.9)	0.312	0.4 (0.1-1.6)	0.193	1.4 (0.4-4.8)	0.574
	3		52	50.0% (35.8-64.2)	19.2% (9.6-32.5)		21.2% (11.1-34.7)		9.6% (3.2-21.0)	
					1.4 (0.5-3.8)	0.544	1.5 (0.6-4.1)	0.397	1.0 (0.3-3.6)	0.946
	4		51	41.2% (27.6-55.8)	27.5% (15.9-41.7)		11.8% (4.4-23.9)		19.6% (9.8-33.1)	
					2.7 (1.0-7.5)	0.070	0.8 (0.3-2.7)	0.748	2.4 (0.8-7.3)	0.118
	5 (most deprived)		72	55.6% (43.4-67.3)	16.7% (8.9-27.3)		16.7% (8.9-27.3)		11.1% (4.9-20.7)	
					Ref		Ref		Ref	
School	School A	9	47	42.6% (28.3-57.8)	23.4% (12.3-38.0)	0.396	19.1% (9.1-33.3)	0.467	14.9% (6.2-28.3)	0.777
					0.6 (0.2-2.1)		1.6 (0.5-5.1)		0.8 (0.2-2.8)	
	School B		51	49.0% (34.8-63.4)	21.6% (11.3-35.3)	0.282	13.7% (5.7-26.3)	0.296	15.7% (7.0-28.6)	0.663
					0.4 (0.1-2.1)		2.7 (0.4-17.3)		0.7 (0.1-3.7)	
	School C		52	42.3% (28.7-56.8)	44.2% (30.5-58.7)	0.307	7.7% (2.1-18.5)	0.199	5.8% (1.2-15.9)	0.069
					0.4 (0.1-2.2)		0.3 (0.0-2.0)		0.1 (0.0-1.2)	
	School D		166	51.8% (43.9-59.6)	18.1% (12.5-24.8)		15.7% (10.5-22.1)		14.5% (9.5-20.7)	
					Ref		Ref		Ref	
Parental knowledge of consumption	Yes	3	256	48.5% (42.3-54.7)	21.8% (16.9-27.2)	0.007	16.0% (11.8-21.0)	0.457	13.7% (9.8-18.5)	0.629
					Ref		Ref		Ref	
	No		54	48.1% (34.3-62.2)	33.3% (21.1-47.5)		7.4% (2.1-17.9)		11.1% (4.2-22.6)	
					3.6 (1.4-9.3)		0.6 (0.2-2.3)		1.4 (0.4-4.8)	
Total			316	48.4% (42.8-54.1)	23.7% (19.2-28.8)		14.6% (10.9-18.9)		13.3% (9.7-17.5)	

P values for the univariate analyses are as follows: gender P = 0.001, age P = 0.081, deprivation P = 0.318, school P = 0.036, parental knowledge p = 0.168. * Provision of alcohol by parents represents the reference category in the outcome variable of the multinomial regression. Ref refers to the reference category of the predictor variable.

Of children who reported consuming alcohol, 25.8% had been drunk at least once in the past month (Table 4). Of those who consumed alcohol without their parent’s knowledge, 11.3% had been drunk at least once in the past month. Children who drank alcohol without their parent’s knowledge were significantly less likely to report past month drunkenness than those who consumed with parental knowledge (29.4% compared with 11.3%; P = 0.002). However, once confounding factors (age, gender, deprivation, and school) had been accounted for, the relationship between past month drunkenness and parental knowledge of consumption was no longer significant (P = 0.082).

**Table 4:** Experiences of drinking children (aged 11-17 years) using chi square and binary logistic regression^*^

		**df**	**n**	**Child reports past month drunkenness**		**Parents report recent talk with child about alcohol (in the last three months)**		**Parents report lifetime use of at least one other parenting technique**	
				**% (95% CI)**	***X***^**2 **^**P**	**% (95% CI)**	***X***^**2 **^**P**	**% (95% CI)**	***X***^**2 **^**P**
				**AOR (95% CI)**	**AOR P**	**AOR (95% CI)**	**AOR P**	**AOR (95% CI)**	**AOR P**
Gender	Male	1	256	27.6% (22.1-33.6)	0.302	94.9% (91.5-97.3)	0.565	46.9% (40.6-53.2)	0.543
				Ref	0.715	Ref	0.375	Ref	0.551
	Female		128	22.1% (15.1-30.5)		90.5% (84.5-94.7)		46.9% (38.0-55.9)	
				0.8 (0.3-2.2)		1.9 (0.4-9.0)		1.3 (0.6-3.0)	
Age	11-14	1	148	17.6% (11.6-25.1)	0.006	90.5% (90.2-96.7)	0.193	34.5% (26.8-42.7)	<0.001
				Ref	0.005	Ref	0.852	Ref	0.058
	15-17		236	30.7% (24.9-37.1)		94.0% (90.2-96.7)		54.7% (48.1-61.1)	
				2.4 (1.3-4.4)		0.9 (0.3-2.7)		1.8 (1.0-3.1)	
Deprivation quintile	1 (most affluent)	4	38	21.6% (9.8-38.2)	0.248	89.2% (74.6-97.0)	0.645	60.5% (43.4-76.0)	0.468
				Ref	0.344	Ref	0.701	Ref	0.538
	2		51	36.7% (23.4-51.7)		94.1% (83.8-98.8)		45.1% (31.1-59.7)	
				1.5 (0.5-4.6)		1.9 (0.3-10.8)		0.5 (0.2-1.3)	
	3		59	21.4% (11.6-34.4)		88.1% (77.1-95.1)		47.5% (34.3-60.9)	
				0.7 (0.2-2.1)		0.9 (0.2-4.5)		0.6 (0.2-1.5)	
	4		61	28.8% (17.8-42.1)		93.4% (84.1-98.2)		55.7% (42.4-68.5)	
				1.0 (0.3-3.1)		1.8 (0.3-10.0)		0.8 (0.3-2.1)	
	5 (most deprived)		82	20.5% (12.2-31.2)		93.9% (86.3-98.0)		46.3% (35.3-57.7)	
				0.7 (0.2-2.0)		2.0 (0.4-10.7)		0.6 (0.2-1.5)	
School	School A	3	62	28.8% (17.8-42.1)	0.478	93.5% (84.3-98.2)	0.400	45.2% (32.5-58.3)	0.034
				Ref	0.259	Ref	0.912	Ref	
	School B		67	24.2% (14.2-36.7)		87.9% (77.5-94.6)		31.3% (20.6-43.8)	0.581
				2.1 (0.7-6.6)		1.1 (0.2-5.8)		1.1 (0.4-2.9)	
	School C		65	18.8% (10.1-30.5)		92.3% (83.0-97.5)		50.8% (38.1-63.4)	
				0.7 (0.3-1.8)		0.7 (0.1-5.2)		1.3 (0.5-3.7)	
	School D		190	28.0% (21.6-35.1)		94.2% (89.8-97.1)		51.6% (44.2-58.9)	
				0.9 (0.4-1.9)		1.4 (0.4-5.4)		1.7 (0.8-3.7)	
Parental knowledge of consumption	Yes	1	307	29.4% (24.3-34.9)	0.002	94.4% (91.3-96.7)	0.008	51.5% (45.7-57.2)	<0.001
				Ref	0.082	Ref	0.045	Ref	0.023
	No		77	11.3% (5.0-21.0)		85.5% (75.6-92.5)		28.6% (18.8-40.0)	
				0.5 (0.2-1.1)		0.4 (0.1-1.0)		0.5 (0.2-0.9)	
Total			384	25.8% (21.3-30.6)		92.7% (89.6-95.1)		46.9% (41.8-52.0)	

^*^ The reference categories are no past month drunkenness reported by the child, no recent alcohol talks reported by the parent, no lifetime use of other parenting technique reported by the parent. Ref refers to reference category.

Parents were asked to report the actions they performed to prevent/reduce alcohol consumption and/or the risks associated with alcohol consumption. Of parenting techniques reported, talking to their child was the most common method used. The reported prevalence of lifetime use of parenting methods to address alcohol use differed between the parents reporting and their child’s reporting. Of dyads where the child reported consuming alcohol, 92.7% of parents reported that they had spoken to their child about alcohol at least once in the past three months, whereas only 57.3% of their children reported this had occurred. However, nearly half (46.9%) of parents of drinking pupils reported having used at least one other parenting approach in the past, compared with 54.2% of their children concurring. Parenting approaches used in the past by parents of drinkers (as reported by parents) included: ensuring their child has a mobile phone (31.0%); offering their child small amounts of alcohol to drink at home (25.0%); organising lifts to and from parties (23.7%); providing the child with alcohol themselves (14.3%); grounding or removing privileges (11.2%); controlling their pocket money or income (8.6%); stopping their child from seeing certain friends (5.7%); reducing their own drinking (5.7%); hiding or reducing alcohol in the house (2.6%); and other (undefined; 1.0%). Children who consumed alcohol without the knowledge of their parents had a significantly lower odds of reporting that their parent had talked to them about alcohol in the past three months, or to report any previous parenting approach, compared with children whose parents were aware of their alcohol consumption (recent talk: AOR 0.4, 95% CI 0.1-1.0; lifetime other parenting technique: AOR 0.5, 95% CI 0.2-0.9).

## Discussion

Our study provides evidence that while drinking is less common in young adolescents (aged 11–14 years), a significant proportion of this age group in our sample who drank alcohol did so without parental knowledge of their actions. Thus, whilst 24.8% of 11–14 year olds reported drinking, 35.1% of this young age group who drank alcohol did so without parental knowledge. This compares with 10.6% parental non-awareness among the 69.6% of children aged 15–17 years who drank alcohol. A comparable study in New York City (USA) identified that 22% of female sixth graders (11–12 years; n = 709) reported consuming alcohol in the past year; with almost none (<1%) of their parents having any knowledge of this [25]. Statistical modelling of our data found that children aged 11–14 years had over a two-fold higher odds of their parent(s) not being aware of their alcohol consumption compared with 15–17 year olds. In the Netherlands, a study of 428 families (with two children) reported that parents were more likely to be aware of the older child’s alcohol consumption than that of the younger child [27]. The authors postulated that this might be because parents become more aware of their child’s alcohol consumption as the child started to drink more regularly with increasing age. It may also be linked to reduced strictness on rules around alcohol consumption as the child gets older [33], as there could be fewer negative consequences for the child revealing their alcohol consumption over time. Our study did not evaluate differences between siblings, with a sole focus on the one child present at the parent–child school meeting on the study day, so we were unable to examine this effect in our sample. Past UK studies also show a strong correlation between frequency and quantity of alcohol consumed and age [5,11,18]. The literature stresses the association between early alcohol initiation and harm [15,16] and a trend towards younger-aged alcohol initiation [3]. It is thus essential that parenting strategies begin while the child is young, and that parents do not assume their child is not at risk of exposure to alcohol. This is particularly important as children may withhold information if they perceive that their parents would disapprove of their alcohol consumption or the friends with whom they plan to be with [30], and because of the value that they may place on privacy [30]. Falsification of identification documents by underage young people to purchase alcohol illustrates the importance children place on participating in adult culture [19].

The most prevalent main method of obtaining alcohol among children was from their parents and through their friends (supporting previous studies [18,19]). Our data found that children who consumed alcohol without parental knowledge had over a three-fold higher odds of obtaining alcohol through their friends than from their parents, compared with children who consumed alcohol with parental knowledge. Whilst this may seem like an obvious observation, further research is required to see the extent to which children are given alcohol by the parental figure or whether alcohol is obtained from the parents without parental knowledge (for example, theft of alcohol stored in the family home). That the absence of parental knowledge was associated with obtaining alcohol from sources other than the parents harbours an important public health message of educating parents not to make alcohol available to other families’ children without the express consent of the parents. Indeed, in countries such as the USA, providing alcohol to minors is a criminal offence (although some States have exceptions such as provision by parents) [34], and this includes parents of underage children’s friends.

Qualitative research in England suggests that the more time young people spend with their friends (friends generally, rather than alcohol-consuming specifically), the more likely they are to drink higher volumes of alcohol (here, defined as drinking seven or more units in one session; one unit equalling 10 ml or 8 g of pure alcohol) [18], and that the most popular location for getting drunk for 14–17 year olds is at a friend’s house (when parents are absent) [9]. This represents a cause for concern since, typically, young people presenting to hospital for alcohol intoxication drink in their friend’s house prior to admission [13]. Communicating with other parents [26] and monitoring children’s friendships [30] are key components in addressing these issues. However, further work is required to understand the most effective ways of doing this.

Talking to their child about alcohol was the most common parenting technique (reported by 92.7% of parents and 57.3% of children). Such discussions may be typically initiated following an individual close to the family having been observed as affected by alcohol (such as the parents, another family member or friends) [26]. Parents may focus on the need for moderation but also cover safety, the law and health implications. Children may welcome these discussions, valuing the discussion around why a rule is being enforced or why certain advice is given [30]. Our data suggest that children whose parents were unaware that they consumed alcohol were significantly less likely to report that their parent talked to them about alcohol in the past three months. This was echoed in the prevalence of the use of other parenting techniques. This reflects published evidence: where parents of female children underestimated risk, the likelihood of parental risk monitoring and communication was significantly lower than where the parent was aware of the child’s behaviour (measures incorporated both alcohol and sexual health) [25]. Similarly, in families where alcohol was a topic of discussion and rules about the situations in which children were allowed to drink were enforced, parents had higher awareness of their child’s alcohol experiences [27]. As with previous research [28], the quality and topic of these conversations was not disclosed, and is worthy of further exploration. Education around alcohol consumption can be included within family and/or school-based interventions (although engaging parents can be problematic) [35,36]. While good communication with children has been associated with lower levels of adolescent alcohol-related harms [28], research should explore the quality and topic of this communication further [28,29]. Our research emphasises the wide range of parenting techniques employed to reduce alcohol consumption and related harms, which go beyond communication (such as ensuring their child has a mobile phone, offering their child small quantities of alcohol to consume at home and organising lifts to and from parties). The cross-sectional nature of this survey prevented us from assessing whether these measures were effective in reducing alcohol consumption and/or related harm. Whilst other parenting strategies such as frequent adolescent monitoring have been associated with increased resistance to alcohol use [37], further research is required to understand the effectiveness of the different parenting strategies that are being employed.

Using a novel methodology of approaching parents and their children attending school events, the survey was able to recruit a large number of dyads. However, the study has a number of limitations. Survey methodologies typically under-report alcohol consumption [38]. This may have been further affected by the practicalities of this survey in that the parent–child dyads, whilst separated, completed the survey in the same room. Young people did report alcohol consumption unknown to their parent(s) but estimates of consumption and of drunkenness may be an underestimate. Further, we cannot be sure if there was an age-related bias in perceptions of drunkenness. To ensure the survey did not impact on the school event and to enable self-completion by young people, the questionnaire was necessarily brief, straightforward and age-appropriate. However, the brevity of the questionnaire limited our ability to analyse the detailed circumstances surrounding young people’s acquisition and consumption of alcohol. Elsewhere, studies have surveyed dyads at different times and/or settings [23,25,29,39]. Although these methods ensured privacy, they did allow discussions about the questionnaire between data collection points and also reduced the potential for linking dyad questionnaires [39]. The survey was not a representative randomised sample and was a convenience sample, based in an area with high levels of alcohol misuse amongst young people [12], limiting generalisability. Random selection of schools approached was not possible because a number of schools refused to participate. The reasons given were: first, that the contemporaneous approach of surveying both child and parent could interfere with school activities; and second, because questioning about alcohol and harms was deemed inappropriate. The participation rate obtained from the surveyed schools (90.0%) was considerably higher than similar surveys [25,28-30,39,40]. Only families with at least one parent present with their child at the school event were recruited. Thus, not all attendees were eligible to take part and families with parents who were disengaged from the school, or with families where children did not attend the event were under-represented. While our survey was unable to explore the experiences of vulnerable groups, it reflected the experiences of ‘ordinary’ families (including 35.4% resident in the lowest UK deprivation quintile) and will inform practice generally [30]. We acknowledge that the sample of children was skewed towards a younger and more male population, which was controlled for in multivariate analyses. Importantly, over-representation of younger children allowed a sample size sufficient to evaluate alcohol consumption and parental knowledge among these younger children.

## Conclusions

In conclusion, the findings from our study suggest that whilst only small numbers of young adolescents in our sample were drinking alcohol, those who did were more likely to do so without their parents’ knowledge. These two factors combined (drinking earlier and drinking without parental knowledge) could place children at risk of immediate harm. Further research is essential to identify whether public health strategies should be developed which could support parents to employ lifestyle parenting techniques even before the parent believes the child to be at risk. Communicating with other parents and monitoring the child’s friendships would be a valuable component of any such strategy developed. However, further research is also required to understand the most effective way of preventing alcohol harm and communicating within the family.

## Abbreviations

IMD: Index of Multiple Deprivation; LSOA: Lower super output area; SES: Socio-economic status.

## Competing interests

The authors have no competing interests to declare.

## Authors’ contributions

MM led the analysis and interpretation and produced the first draft of the paper. PPH, MM and PC contributed to the questionnaire design. PC and PPH contributed to analysis, interpretation and refining the paper. GE co-ordinated the survey, input the data and helped to draft the paper but sadly died before the final version was complete. GE read and approved a near final version of the paper. MM, PPH and PC read and approved the final manuscript.

## Pre-publication history

The pre-publication history for this paper can be accessed here:

http://www.biomedcentral.com/1471-2458/13/412/prepub
